# MCEENet: Multi-Scale Context Enhancement and Edge-Assisted Network for Few-Shot Semantic Segmentation

**DOI:** 10.3390/s23062922

**Published:** 2023-03-08

**Authors:** Hongjie Zhou, Rufei Zhang, Xiaoyu He, Nannan Li, Yong Wang, Sheng Shen

**Affiliations:** 1 School of Automation, Central South University, Changsha 410083, China; 2 Beijing Institute of Control and Electronic Technology, Beijing 100038, China

**Keywords:** few-shot semantic segmentation, multi-scale context enhancement, edge-assisted segmentation

## Abstract

Few-shot semantic segmentation has attracted much attention because it requires only a few labeled samples to achieve good segmentation performance. However, existing methods still suffer from insufficient contextual information and unsatisfactory edge segmentation results. To overcome these two issues, this paper proposes a multi-scale context enhancement and edge-assisted network (called MCEENet) for few-shot semantic segmentation. First, rich support and query image features were extracted, respectively, using two weight-shared feature extraction networks, each consisting of a ResNet and a Vision Transformer. Subsequently, a multi-scale context enhancement (MCE) module was proposed to fuse the features of ResNet and Vision Transformer, and further mine the contextual information of the image by using cross-scale feature fusion and multi-scale dilated convolutions. Furthermore, we designed an Edge-Assisted Segmentation (EAS) module, which fuses the shallow ResNet features of the query image and the edge features computed by the Sobel operator to assist in the final segmentation task. We experimented on the PASCAL-$5^{i}$ dataset to demonstrate the effectiveness of MCEENet; the results of the 1-shot setting and 5-shot setting on the PASCAL-$5^{i}$ dataset are 63.5% and 64.7%, which surpasses the state-of-the-art results by 1.4% and 0.6%, respectively.

## 1. Introduction

**Figure 1:**
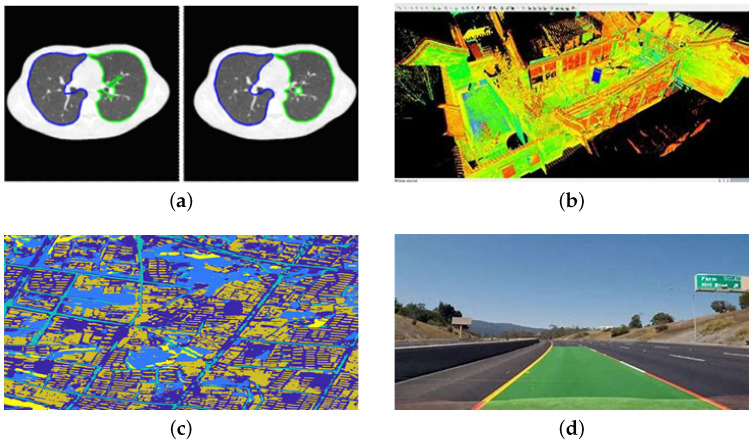
Examples of areas where semantic segmentation can be used. (**a**) Medical image; (**b**) 3D point clouds image; (**c**) Remote sensing image; (**d**) Lane mark detection image.

As a fundamental problem in the field of computer vision, semantic segmentation has obtained tremendous improvements during the past few years. As shown in Figure 1, it has been widely used in medical image recognition [1], 3D points Clouds [2], geological exploration [3], cloud and cloud shadow segmentation [4,5], remote sensing image [6,7,8,9], and automatic driving [10], etc. Existing semantic segmentation models based on convolutional neural networks (CNNs) (e.g., U-Net [11] and DeepLab [12]) often rely on a large amount of pixel-level labeled data, which leads to the following two problems: (1) it costs plenty of labor due to the fact that all training samples should be marked manually one by one, and (2) these models perform poorly in recognizing novel objects. The above challenges limit the application of semantic segmentation models. Therefore, the research on few-shot semantic segmentation (FSS) has become one of the most urgent and crucial tasks in computer vision [13].

Existing FSS methods can be divided into single-prototype FSS methods [14,15] and multi-prototype methods [16,17] according to the structure of the prototypical network [18]. Specifically, single-prototype FSS methods usually encode support and query images into a high-dimensional space through CNNs to obtain their features. Then, a masked average pooling strategy is adopted to calculate a single prototype from the features of support images. Afterward, some distance measurement methods (e.g., cosine similarity [19] and Euclidean distance) are applied to measure the distances between query features and a single prototype in the high-dimensional space. Finally, the measured distance is used to distinguish the foreground and background of query images to acquire their segmentation prediction maps. Different from single-prototype FSS methods, multi-prototype FSS methods improve the prototype structures of single-prototype ones. They obtain a good segmentation performance by computing multiple prototypes of each target class. However, multi-prototype FSS methods can only generate partial prototypes of support features [17], which leads to a lack of important local information about the target class. Therefore, the generalization performance of these methods is poor when facing a new object.

To address this issue, researchers have proposed some adaptive learning-based FSS methods that exploit adaptive convolutional structures to learn important local information about target classes [20]. First, they use CNNs to obtain support and query image features. Then, these image features together with support masks are further processed using convolutional structures with learnable parameters. Finally, the processed features are upsampled to obtain the final segmentation result. However, the above methods suffer from insufficient contextual information due to their simple convolutional structures. In semantic segmentation, contextual information provides pixel information around objects, which is extremely critical for semantic segmentation performance. Furthermore, the above methods tend to ignore the edges of the input image. For some input cases with complex or blurred edges, these methods will inevitably produce poor edge segmentation results.

To overcome the above two issues, we propose a multi-scale context enhancement and edge-assisted network (called MCEENet) for FSS. In MCEENet, we first build two weight-shared feature extraction networks to extract support and query image features, respectively. Each feature extraction network consists of a ResNet-50 and a Vision Transformer, where ResNet-50 extracts local image features and Vision Transformer captures global dependencies of the image. After each feature extraction network, we propose a multi-scale context enhancement (MCE) module to fuse and refine ResNet and Vision Transformer features. Additionally, we design an edge-assisted segmentation (EAS) module, which fuses the shallow ResNet features of the query image and the edge features computed by the Sobel operator to generate the edge guidance feature. The main contributions of this paper can be summarized as follows:

- We proposed two MCE modules to enhance the contextual information of the support and query image features. Each MCE module first concatenates the ResNet-50 and Vision Transformer features and employs pooling operations with different pooling rates to generate multi-scale features. Then, it fuses the features of adjacent scales through cross-scale feature fusion, and uses multi-scale dilated convolutions to mine and enrich the contextual information of the fused features;
- We designed an EAS module to improve edge parts of the segmentation results. The EAS module combines the shallow features of the query image extracted by ResNet-50 (including details of objects) with the edge features calculated by the Sobel operator (including boundaries of objects) to generate an edge guidance feature. Subsequently, this edge guidance feature was used as a clue for segmentation prediction, thereby improving edge details in FSS;
- The effectiveness of MCEENet was demonstrated on the PASCAL-5${}^{i}$ dataset. The comparative results suggest that MCEENet achieves superior semantic segmentation performance compared with state-of-the-art methods for FSS.

The rest of this paper is organized as follows. Section 2 introduces the related work. The detailed architecture and main components of MCEENet are elaborated in Section 3. Extensive experimental studies are carried out in Section 4 to demonstrate the effectiveness of MCEENet. Finally, Section 5 concludes this paper.

## 2. Related Work

Existing semantic segmentation methods are mostly based on fully convolutional networks (FCNs) [21], using operations such as convolution, upsampling, and skip connections to build an end-to-end semantic segmentation network. Later, pyramid scene parsing network [22] utilizes pyramid pooling module and dilated convolution to integrate contextual information from different scales to obtain a large receptive field. U-Net [11] extracts low-level and high-level information of the input image by connecting features at different levels using an encoder-decoder structure. DeepLab-V3 [23] and DeepLab-V3+ [24] introduce operations such as dilated convolution, fully connected conditional random fields (CRFs), and dilated spatial pyramid pooling (ASPP) to improve segmentation performance. Although these methods have made some progresses in semantic segmentation, they usually cost plenty of labor due to the fact that all training samples require pixel-level annotations. Moreover, they cannot generalize to novel objects. Therefore, some researchers have tried to investigate FSS methods.

FSS methods [16,25,26,27,28] aim at providing dense segmentation results for new class query images with only few labeled support images. Shaban et al. [13] developed a pioneering work named OSLSM, where support images are used to generate classifier weights for query image predictions. Wang et al. [14] proposed an FSS network with prototype alignment called PANet. PANet makes full use of the knowledge of support images, and uses cosine distance for final segmentation. Gairola et al. [29] proposed a novel similarity propagation network, which finds that the background region of different images from the same class have strong similarity, and uses this similarity to improve segmentation performance. Zhang et al. [30] proposed a similarity guidance network (SG-One), which uses masked average pooling to extract foreground and background features of support images. In [15], an improved feature weighting and boosting network based on SG-One is developed. This network introduces a regularization term when calculating cosine similarity, which enhances the activation values of foreground features and suppresses the activation values of background features, thereby improving the discriminative ability of the network. Unfortunately, the above FSS methods use only a single prototype to represent the class center of support images in the high-dimensional space. Their segmentation performance is challenged for objects with dramatic appearance changes and scene changes.

To overcome this shortcoming, researchers have proposed a series of multi-prototype FSS methods [16,19]. Liu et al. [17] proposed a part-aware network based on attention mechanism, which uses simple linear iterative clustering to segment images from the test set to obtain masks of multiple regions, thereby extending a single prototype to multiple prototypes. Li et al. [31] presented an adaptive superpixel-guided network that leverages superpixels to adapt the number and support regions of prototypes, making the prototypes content-adaptive and spatially aware. Yang et al. [32] observed that the image background may contain class information, and used *k*-means to generate multiple local prototypes for joint training. In [16], a prototype mixture model is proposed to associate various image regions with multiple prototypes using expectation maximization, which enriches prototype-based semantic representations. Fan et al. [19] designed a self-support prototype network. This network uses a traditional prototype matching algorithm to extract self-support prototypes on the query image, and then fuses the self-support prototypes with initial support prototypes to improve segmentation performance. Although achieving better performance than single-prototype FSS methods, these multi-prototype FSS methods lose important local information and thus generalize poorly to new objects.

To address this problem, some FSS methods based on adaptive learning [33,34,35,36,37] have been proposed to learn important local information of target classes through adaptive convolutional structures. For instance, Zhang et al. [38] proposed a class-agnostic segmentation network based on masked average pooling, which designs an iterative update strategy to optimize the decoder to refine the segmentation result. Tian et al. [39] developed a feature enrichment module to integrate multi-scale context information to improve segmentation performance. In [40], a self-guided and cross-guided learning network is proposed to supplement the lost information caused by masked average pooling operation. A novel cross-reference network is proposed in [41], which finds common features in support and query images and utilizes these common features to facilitate the FSS task. Yang et al. [42] designed an information exchange module to activate the common features of the similar parts between support and query images. Xie et al. [43] proposed a self-attention mechanism to enrich the multi-scale features of support and query images. Despite prevalence, the above adaptive learning-based FSS methods suffer from insufficient contextual information due to their simple convolution structures. Moreover, these methods tend to ignore the edges of the input image, leading to poor edge segmentation results. To solve these two issues, we propose a novel FSS approach named MCEENet, which is described in detail next.

## 3. Methodology

### 3.1. Problem Definition

The key difference between FSS and general semantic segmentation is that the classes in training and test sets of FSS are not related. This means that in the test stage of FSS, the test set has classes that are completely unseen in the training stage. Existing methods mainly use the meta-learning paradigm to train models, during which the models are expected to learn sufficient transferable knowledge on the meta-training dataset (denoted as $D_{train}$) and show good segmentation performance on the meta-test dataset (denoted as $D_{test}$) with few labeled samples. In particular, $D_{train}={\left\{\begin{array}{c} \left(I_{i},M_{i}\right) \end{array}\right\}}_{i=1}^{N_{train}}$ is composed of $N_{train}$ image-mask pairs for training and $D_{test}={\left\{\begin{array}{c} \left(I_{i},M_{i}\right) \end{array}\right\}}_{i=1}^{N_{test}}$ consists of $N_{test}$ image-mask pairs for test. Herein, $I_{i}$ indicates the *i*th image and $M_{i}$ is its corresponding mask. Note that object classes in $D_{train}$ and $D_{test}$ are not related to each other, i.e., $D_{train}\cap D_{test}=⌀$.

We adopt the standard FSS settings [39,44,45]. Specifically, in the episodic training and test stages, we randomly sample from $D_{train}$ and $D_{test}$ to form a set of training episodes $E_{train}={\left\{\begin{array}{c} \left(S_{i},Q_{i}\right) \end{array}\right\}}_{i=1}^{N_{train\_ep}}$ and test episodes $E_{test}={\left\{\begin{array}{c} \left(S_{i},Q_{i}\right) \end{array}\right\}}_{i=1}^{N_{test\_ep}}$, respectively, where $N_{train\_ep}$ and $N_{test\_ep}$ are the numbers of training and test episodes. Each training/test episode contains a small support set S and a small query set Q. Specifically, $S={\left\{\begin{array}{c} \left(I_{i}^{s},M_{i}^{s}\right) \end{array}\right\}}_{i=1}^{K}$ is composed of K support image-mask pairs of the same class and $Q=\left\{\begin{array}{c} \left(I^{q},M^{q}\right) \end{array}\right\}$ represents a query image-mask pair of the same class as S. In each training episode, the model predicts the segmentation mask (denoted as ${\hat{M}}^{q}$) of $I^{q}$ by learning the mapping of image-mask pairs in S. Afterward, the binary cross-entropy loss (denoted as $BCE({\hat{M}}^{q},M^{q})$) is calculated to update the weights of the model. Once the model is trained completely, we can evaluate the segmentation performance of the model on $E_{test}$.

### 3.2. Architecture Overview

**Figure 2:**
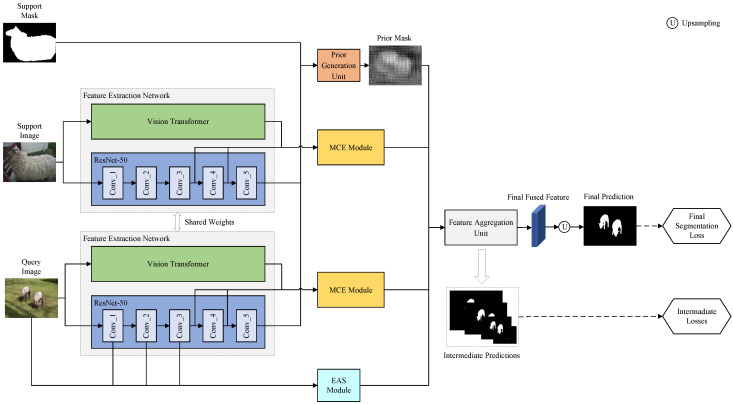
Overall network framework of the proposed MCEENet. The query image and support image are fed into the feature extraction network (weight-shared) to extract middle-level features (inside the larger gray dotted box), green/blue represent ViT/Resnet-50 feature extraction network. The extracted middle-level features then enhanced by the MCE module. The prior generation unit generates the prior mask of the query image using support image, support mask, and query image. The EAS module uses the Sobel operator to obtain the edge guidance feature of query image. Finally, the segmentation results are obtained through a feature aggregation unit and an upsampling unit.

In this work, we proposed a multi-scale context enhancement and edge-assisted network to perform image semantic segmentation under the case of small samples. The backbone of the proposed network is two parallel ViT and ResNet-50 networks, which have excellent performance in extracting image features. The proposed network mainly consists of two parallel weight-shared feature extraction networks, an MCE module, an EAS module, a prior generation unit, a feature aggregation unit, and an upsampling unit. Among these components, the first four are used to extract image features, and the latter are used to fuse these features and generate the final segmentation results. The framework of the proposed network is shown in Figure 2.

In the first step, we extracted four image features. First, two parallel feature extraction networks were used to extract support and query image features, respectively. Each feature extraction network was composed of a ResNet-50 extracting local image features and a Vision Transformer capturing global dependencies of the image, which we will describe in detail in Section 3.3. Note that we loaded pretrained weights on ImageNet for both ResNet-50 and Vision Transformer. Then, after each feature extraction network, we built an MCE module to fuse and further enhance support and query image features extracted by ResNet-50 and Vision Transformer, which we will describe in detail in Section 3.4. Afterward, with the aim of learning robust object edges, an EAS module was used for fusing the shallow ResNet features of the query image and the edge features computed by the Sobel operator to generate the edge guidance feature, which will be introduced in Section 3.5. Additionally, by using high-level ResNet features of the support and query images and support mask, the prior generation unit employed a training-free distance metric method to generate the prior mask for each query image.

In the second step, we generated segmentation results. The feature aggregation unit was designed to fuse the above four image features: (1) the support image features enhanced by the MCE module, (2) the query image features enhanced by the MCE module, (3) the edge guidance feature generated by the EAS module, and (4) the prior mask output generated by the prior generation unit. The fused feature output by the feature aggregation unit was sent to the upsampling unit to produce the final segmentation result. It should be noted that the designs of the prior generation unit and the feature aggregation unit were the same as those in [39]. In the following, we describe the main components of MCEENet in detail.

### 3.3. Feature Extraction Networks

Regarding existing FSS methods, most of them only employ CNNs (e.g., VGG [46] or ResNet-50 [47]) to extract features of support and query images. Although these methods can obtain meaningful local features for FSS tasks, they cannot model the global relationships of images. Recently, Vision Transformers with powerful global self-attention ability for capturing global dependencies have emerged and achieved good performance in a range of computer vision tasks [48]. Inspired by this, we designed two weight-shared feature extraction networks to extract support and query features, respectively, each of which was composed of a ResNet-50 and a Vision Transformer. As shown in Figure 2, for an input support or query image, we employed a parallel structure of ResNet-50 and a Vision Transformer to extract local and global features of the image, respectively. Next, we briefly introduce the architectures of ResNet-50 and Vision Transformer.

(1) *ResNet-50*: ResNet-50 is composed of five groups of convolutional layers, denoted as conv_1, conv_2, conv_3, conv_4, and conv_5, respectively. As shown in Figure 2, we denote the output feature maps of conv_1, conv_2, conv_3, conv_4, and conv_5 as $F_{1}$, $F_{2}$, $F_{3}$, $F_{4}$, and $F_{5}$, respectively. Suppose that an image with three channels is input to ResNet-50, the channels of $F_{1}$, $F_{2}$, $F_{3}$, $F_{4}$, and $F_{5}$ are 64, 256, 512, 1024, and 2048, respectively, and their corresponding resolutions are 1/4, 1/4, 1/8, 1/16, and 1/32 of the original image size, respectively. Specifically, conv_1 contains a 7×7 convolutional layer and a max pooling layer, and conv_2, conv_3, conv_4, and conv_5 are stacked by residual blocks. A residual block is stacked by multiple convolutional, batch normalization, and ReLU activation layers. Assuming that the input of the residual block is *x*, the output *y* of the residual block is calculated as follows:

(1) y=f(x,w)+x,

where f(·) denotes the residual mapping formed by the stacked layers, and *w* denotes the parameters of these layers. It can be seen from Equation (1) that the input signal can be directly sent to the output of the residual block, so the gradient vanishing problem can be addressed. Moreover, due to the nature of convolutions, ResNet-50 can learn meaningful local features of images with the help of residual blocks.

(2) *Vision Transformer*: The Vision Transformer consists of a patch and position embedding layer, a Transformer encoder, and a classification head. In the patch and position embedding layer, the Vision Transformer first splits the input image evenly into a series of patches. Then, these patches are flattened and projected into a *D*-dimensional vector (called patch embedding), and a learnable class embedding is added to the head of the patch embedding to represent the whole image. To preserve the location information of the patches, a location embedding is added to the patch embedding. Afterward, this combined embedding is sent to the Transformer encoder for feature extraction. Specifically, the Transformer encoder consists of alternating multi-head self-attention (MSA) blocks and multi-layer perceptron (MLP) blocks. We denote the input of an MSA module as $z\in N^{N\times D}$, where *N* is the number of tokens. Note that *z* contains the information of all patches of the input image. Then, *z* is transformed to queries $Q\in N^{N\times D^{{}^{\prime }}}$, keys $K\in N^{N\times D^{{}^{\prime }}}$, and values $V\in N^{N\times D^{{}^{\prime }}}$ through linear transformations with different weights, where $D^{{}^{\prime }}$ denotes the *Q*-*K*-*V* dimension. Next, the self-attention operation is calculated as follows:

(2) $$Attention\left(Q,K,V\right)=softmax\left(\frac{QK^{T}}{\sqrt{m}}\right)V,$$

where softmax(·) denotes the softmax activation operation and $\frac{1}{\sqrt{m}}$ is the scaling factor. From Equation (2), we calculate the correlations of each element with the other elements in the sequence, i.e., model the global dependencies of the image. Therefore, the Vision Transformer with self-attention mechanism is capable of integrating global information of the image. Finally, the classification head receives the output of the Transformer encoder for final classification. Note that the proposed MCEENet uses the structures before the final classification of the Vision Transformer.

### 3.4. MCE Module

Existing FSS methods usually use simple convolutional structures as the backbone and thus suffer from insufficient contextual information. In semantic segmentation, contextual information plays an important role in segmentation performance, because it provides rich pixel information around objects. To this end, we designed the MCE module after each feature extraction network, with the aim of further mining and enriching the contextual information of the extracted features.

**Figure 3:**
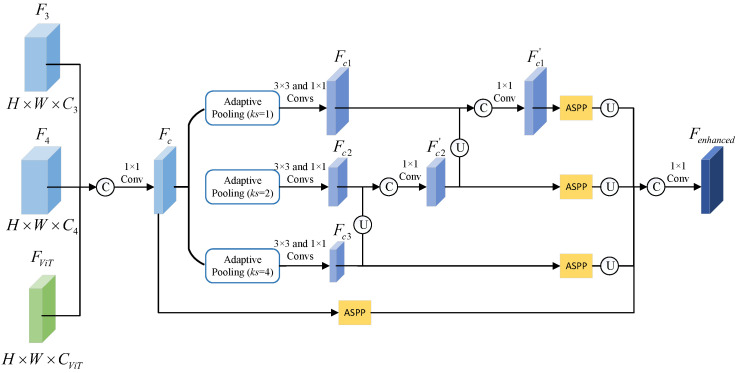
The visual illustration of the MCE module, which receives two ResNet-50 features and one ViT feature, and then uses multi-scale pooling operations with different pooling rates and four parallel ASPP modules to generate enhanced features.

The structure of each MCE module is shown in Figure 3. Its input includes $F_{3}$ and $F_{4}$ generated by ResNet-50 and $F_{ViT}$ extracted by Vision Transformer. First, we fuse these three features to generate $F_{c}$ through feature concatenation and the 1×1 convolution:

(3) $$F_{c}=F^{1\times 1}\left(C\left(F_{3},F_{4},F_{ViT}\right),\theta^{1\times 1}\right),$$

where C denotes the concatenation operation and $F^{1\times 1}$ represents the 1×1 convolution with parameter $\theta^{1\times 1}$. Then, $F_{c}$ is processed by multi-scale pooling operations with different pooling rates (i.e., 1, 2, and 4) followed by the 3×3 and 1×1 convolutions, which generates $F_{c1}$, $F_{c2}$, and $F_{c3}$. In order to enhance the feature interaction between adjacent scales, we adopted a bottom-up cross-scale feature fusion. Specifically, $F_{c3}$ is upsampled by 1 time, and it is concatenated with $F_{c2}$ followed by a 1×1 convolution to generate $F_{c2}^{{}^{\prime }}$. By using the same operations, we fuse $F_{c2}^{{}^{\prime }}$ and $F_{c1}$ to generate $F_{c1}^{{}^{\prime }}$. The above cross-scale feature fusion operations can be summarized as follows:

(4) $$F_{c2}^{{}^{\prime }}=F^{1\times 1}\left(C\left(U\left(F_{c3}\right),F_{c2}\right),\theta^{1\times 1}\right),$$

(5) $$F_{c1}^{{}^{\prime }}=F^{1\times 1}\left(C\left(U\left(F_{c2}^{{}^{\prime }}\right),F_{c1}\right),\theta^{1\times 1}\right),$$

where *U* represents the upsampling operation. Afterward, we used four parallel ASPP modules to process $F_{c1}^{{}^{\prime }}$, $F_{c2}^{{}^{\prime }}$, $F_{c3}$, and $F_{c}$, respectively. Herein, each ASPP module consisted of four parallel dilated convolutions with different dilated rates (i.e., 1, 12, 24, and 36), which was used to further encode and capture contextual information. Finally, we concatenated the four features processed by the four ASPP modules followed by a 1×1 convolution to adjust the number of channels, which generated the final output feature, i.e., $F_{enhanced}$.

### 3.5. EAS Module

Edges are very important for semantic segmentation as they describe the shapes and specific contours of objects. Accurate identification of edges can greatly improve the accuracy of semantic segmentation. However, for existing FSS methods, they do not take any measures to deal with the edges of objects, thus suffering from poor edge segmentation results. To solve this problem, we propose the EAS module, which combines the shallow features of CNN with the edge feature calculated by the Sobel operator to learn robust object edges.

**Figure 4:**
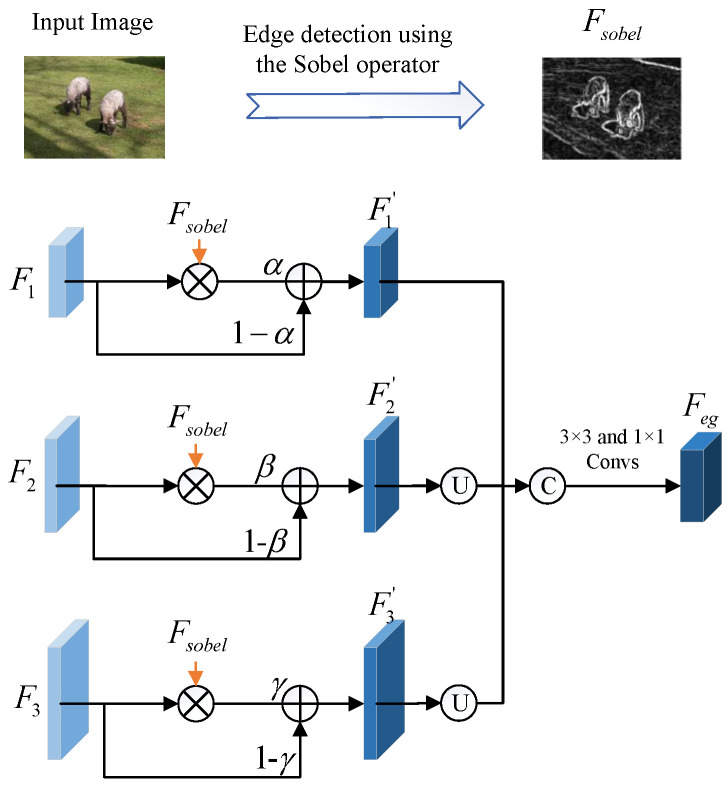
The visual illustration of the EAS module, which receives three shallow ResNet-50 features of the query image, and uses the Sobel operator to generate the edge guidance feature.

The structure of the EAS module is shown in Figure 4, which includes four inputs: the input query image $I^{q}$ and the output features of the first three stages of ResNet-50 (i.e., $F_{1}$, $F_{2}$, and $F_{3}$). First, we use the Sobel operator to perform edge detection on $I^{q}$ followed by the sigmoid normalization to obtain the single channel feature (denoted as $F_{sobel}$):

(6) $$F_{sobel}=S\left(Sobel\left(I^{q}\right)\right),$$

where Sobel represents the Sobel operator and S represents the Sigmoid activation function. Then, we used $F_{sobel}$ as the attention map to refine the edge parts of $F_{1}$, $F_{2}$, and $F_{3}$, respectively. For simplicity, we only introduced the attention operations on $F_{1}$. Specifically, we first downsampled $F_{sobel}$ to the resolution of $F_{1}$ and then multiplied it with $F_{1}$, with the aim of highlighting the responses of the edge parts on it. To ensure the stability of attention learning, we calculated the weighted sum of the attention feature and $F_{1}$ as the final refined feature, namely $F_{1}^{{}^{\prime }}$. Using the same operations, $F_{2}^{{}^{\prime }}$ and $F_{3}^{{}^{\prime }}$ can be obtained. The above attention operations can be summarized as follows:

(7) $$F_{1}^{{}^{\prime }}=\alpha \left(F_{sobel}⊙F_{1}\right)+\left(1-\alpha \right)F_{1},$$

(8) $$F_{2}^{{}^{\prime }}=\beta \left(F_{sobel}⊙F_{2}\right)+\left(1-\beta \right)F_{2},$$

(9) $$F_{3}^{{}^{\prime }}=\gamma \left(F_{sobel}⊙F_{3}\right)+\left(1-\gamma \right)F_{3},$$

where α, β, and γ are the weighting factors representing the contributions of the attention features to the final refined features, respectively. It is worth noting that α, β, and γ are initially set to 0, and their values can be adaptively adjusted during model training. Finally, we upsample $F_{2}^{{}^{\prime }}$ and $F_{3}^{{}^{\prime }}$ to the same size as $F_{1}^{{}^{\prime }}$, and fuse them through concatenation and the 3×3 and 1×1 convolutions to generate the final edge guidance feature, denoted as $F_{eg}$.

### 3.6. Loss Function

Our MCEENet is an end-to-end learning system for FSS tasks. In general, its loss consists of the final segmentation loss produced by the final prediction, and the intermediate segmentation losses generated by the predictions at all spatial scales in the aggregation unit. Similar to [39], we combined these losses as a total loss:

(10) $$L_{total}=\lambda \sum_{i=1}^{N}L_{intermediate}^{i}+L_{final},$$

where $L_{final}$ denotes the final segmentation loss, $L_{intermediate}^{i}$ indicates the intermediate segmentation loss at the *i*th spatial scale, and *N* is the number of spatial scales in the aggregation unit. Note that λ is a weighting factor to adjust the contributions of the intermediate segmentation losses in $L_{total}$. We set λ to 1 in all our experiments to keep the same experimental setting as in [39]. For each of $L_{intermediate}^{i}$ and $L_{final}$, the binary cross-entropy loss was selected to calculate the segmentation loss:

(11) $$L_{bce}=-\frac{1}{n}\sum \left[M^{q}\ln{\hat{M}}^{q}+\left(1-M^{q}\right)\ln\left(1-{\hat{M}}^{q}\right)\right],$$

where $M^{q}$ and ${\hat{M}}^{q}$ represent the ground-truth and predicted query masks, respectively and *n* denotes the number of pixels in the query mask.

## 4. Experimental Studies

### 4.1. Dataset and Evaluation Metrics

The performance of MCEENet was examined on the PASCAL-$5^{i}$ [13] dataset. PASCAL-$5^{i}$ includes images from the PASCAL VOC 2012 [49] and extra annotations from SBD [50]. A total of 20 classes in the PASCAL-$5^{i}$ dataset were evenly divided into four splits for four-fold cross-validation. Specifically, three splits (containing 15 classes) were selected for training and the remaining one (containing five classes) was used for testing. The specific test classes of each split are shown in Table 1.

Two commonly used evaluation metrics were used to compare the performance of MCEENet and other FSS methods, including mean Intersection over Union (mIoU) and foreground-background IoU (FB-IoU). Given a certain class *i*, its IoU is defined as follows:

(12) $$IoU_{i}=\frac{TP}{TP+FP+FN},$$

where *TP*, *FP*, and *FN* denote true positive, false positive, and false negative, respectively. Then, mIoU is calculated by averaging IoUs of all classes:

(13) $$mIoU=\frac{1}{C}\sum_{i=1}^{C}IoU_{i},$$

where *C* denotes the number of classes of the test set. Herein, *C* is 5 when calculating mIoU on the PASCAL-$5^{i}$ dataset. With respect to FB-IoU, it only considers two classes, i.e., the foreground class and the background class, without considering the specific class of each object. Therefore, by setting *C* in Equation (13) to 2, we can calculate FB-IoU, which represents the mean of IoUs of the foreground and background classes.

### 4.2. Experimental Design

In order to avoid model overfitting in the training procedure, we first performed online data augmentation on training images, including random image scaling (0.9–1.1), random rotation (−10${}^{\circ }$ to 10${}^{\circ }$), random Gaussian blur (Gaussian kernel size 5×5), and random horizontal flip. Then, these augmented images were resized to 473×473 and input into the model. The SGD algorithm was used as the optimizer. The initial learning rate was set to 0.0025. The momentum and weight decay were set to 0.9 and 0.0001, respectively. We adopted the poly policy in [45] to decay the learning rate, where power was set to 0.9. The pretrained weights of ResNet-50 and the Vision Transformer on ImageNet were loaded for accelerating the training procedure. The training batch size and the maximum number of training epochs were set to 4 and 200, respectively. As mentioned in Section 4.1, we tested the performance of MCEENet on the PASCAL-$5^{i}$ dataset in a four-fold cross-validation manner, and reported the performance on each split and its average performance.

### 4.3. Ablation Study

MCEENet proposes two new modules for FSS tasks, i.e., the MCE module and the EAS module. The former is designed to enhance contextual semantics and the latter is used to learn robust object edges. To demonstrate the effectiveness of these two modules, we conducted ablation experiments on each of them. In addition, MCEENet not only uses ResNet to extract image features, but also uses an additional Vision Transformer. Therefore, we also tested the effect of the additional Vision Transformer. The results of the ablation experiments are given in Table 2, including mIoU of MCEENet, MCEENet without Vision Transformer, MCEENet without the MCE modules, and MCEENet without the EAS module. Note that the performance of these methods was obtained under the experimental settings of 1-shot and 5-shot.

**Figure 5:**
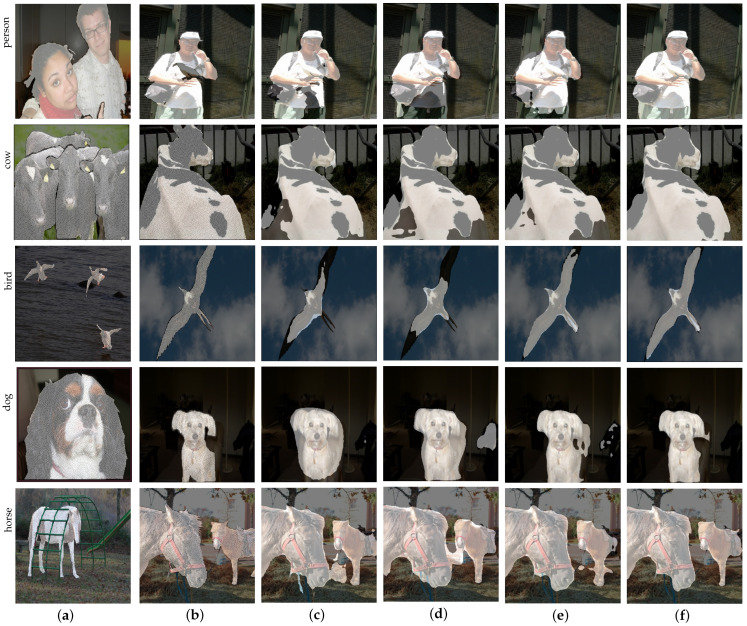
Qualitative ablation results in 1-way 1-shot segmentation on PASCAL-$5^{i}$. Specifically, the first column is the support images with ground-truths, the second column is the query images with ground-truths, and the third, fourth, fifth, and sixth columns are the segmentation results of the query images obtained by MCEENet without Vision Transformer, MCEENet without the MCE modules, MCEENet without the EAS module, and MCEENet, respectively. (**a**) support; (**b**) ground-truth; (**c**) MCEENet without Vision Transformer; (**d**) MCEENet without the MCE modules; (**e**) MCEENet without the EAS module; (**f**) MCEENet.

(1) *Vision Transformer*: In order to verify the effectiveness of the additional Vision Transformer, we compared MCEENet with MCEENet without Vision Transformer. Note that MCEENet without the Vision Transformer was a variant by removing Vision Transformer from MCEENet, i.e., only ResNet-50 was used for feature extraction. In addition, in MCEENet without Vision Transformer, the MCE module only took $F_{3}$ and $F_{4}$ of ResNet-50 as inputs. From Table 2, it can be seen that mIoU of MCEENet without Vision Transformer is 0.9% and 1.5% lower than that of MCEENet under the experimental settings of 1-shot and 5-shot, respectively. The segmentation results in Figure 5 also suggest that the Vision Transformer can improve the segmentation performance of our method in FSS tasks. We attribute this performance improvement to the Vision Transformer’s powerful global feature extraction capability.

(2) *MCE module*: With respect to MCEENet without the MCE modules, we removed the two MCE modules from MCEENet. In order to ensure the validity of the network, we used simple feature concatenation followed by a 1×1 convolution to fuse $F_{3}$, $F_{4}$, and $F_{ViT}$. It can be seen from Table 2 that under the experimental settings of 1-shot and 5-shot, mIoU of MCEENet without the MCE module decreases by 2.2% and 2.1% compared with that of MCEENet, respectively. The segmentation results in Figure 5 also show that removing the MCE modules greatly reduces the performance of MCEENet. The above results suggest that the MCE modules can capture effective context information, which is critical for improving FSS performance.

(3) *EAS module*: For MCEENet without the EAS module, we directly deleted the EAS module in MCEENet. As a result, we did not generate the edge guidance feature, and in the following feature aggregation unit, only the support and query features enhanced by the MCE modules and prior masks were used to generate the final fused feature. From Table 2, under 1-shot and 5-shot experimental settings, mIoU of MCEENet without the EAS module drops by 0.4% and 0.5% compared with that of the counterpart, respectively. From Figure 5, MCEENet can produce more precise edge segmentation results than MCEENet without the EAS module. The above results validate the effectiveness of edge feature learning in our EAS module, which is beneficial for FSS tasks.

### 4.4. Comparison with State-of-the-Art Methods

We also compared MCEENet with other state-of-the-art FSS methods on the PASCAL-$5^{i}$ dataset under 1-shot and 5-shot experimental settings. These state-of-the-art FSS methods can be classified into two classes: (1) methods based on prototype learning: FWB [15], PANet [14], SG-One [30], ASGNet [31] and SAGNN [34] and (2) methods based on adaptive learning: OSLSM [13], CRNet [41], HSNet [51], PFENet [39], CWT [35] and SCLPFENet [40]. The hyperparameter settings used in all these methods are set the same as in Section 4.2. The results of these methods in terms of mIoU and FB-IoU are shown in Table 3 and Table 4, respectively. Note that the performance of the 13 compared methods was derived from their original papers. Moreover, as shown in these two tables, we divided the 13 compared methods into two kinds according to the backbone, i.e., the VGG-16-backbone methods (containing seven methods) and the ResNet-50-backbone methods (containing six methods). Different from these two kinds of methods, MCEENet employed ResNet-50 together with Vision Transformer for feature extraction.

From Table 3, under the 1-shot experimental setting, MCEENet achieves the best mIoU on two out of four folds (i.e., Fold-0 and Fold-1) on the PASCAL-$5^{i}$ dataset, and also obtains the best average mIoU among all the compared methods. Under the 5-shot experimental setting, MCEENet also produces the best mIoU on Fold-0 and Fold-1, and beats all other methods in terms of the average mIoU. Specifically, compared with PFENet [39] which proposes the prior generation and feature aggregation units, MCEENet improves the average mIoU by 2.7% and 2.6% under the experimental settings of 1-shot and 5-shot, respectively. Compared with HSNet [51], which yields the best performance among the seven VGG-16-backbone methods, MCEENet improves the average mIoU by 3.8% and 0.6% under the experimental settings of 1-shot and 5-shot, respectively. Compared with the recently released ResNet-50-backbone method called SAGNN [34], mIoU of MCEENet increases by 1.4% and 1.9% under the experimental settings of 1-shot and 5-shot, respectively. The second best methods on the 1-shot and 5-shot settings are SAGNN [34] and HSNet [51], respectively, they achieve an mIoU of 62.1% and 64.1%, respectively. MCEENet improves the average mIoU by 1.4% and 0.6% under the settings of 1-shot and 5-shot. The above results suggest that MCEENet obtains better semantic segmentation performance than other state-of-the-art FSS methods on the four folds of the PASCAL-$5^{i}$ dataset.

The results in Table 4 show that, among all the compared methods, MCEENet achieves the best FB-IoU of 77.0% and 77.8% under the 1-shot and 5-shot experimental settings, respectively. The second best methods on the 1-shot and 5-shot settings are PFENet [39] and ASGNet [31], respectively, they achieve an FB-IoU of 62.1% and 64.1%, respectively. MCEENet improves the FB-IoU by 3.7% and 3.9% under the 1-shot and 5-shot experimental settings.

**Figure 6:**
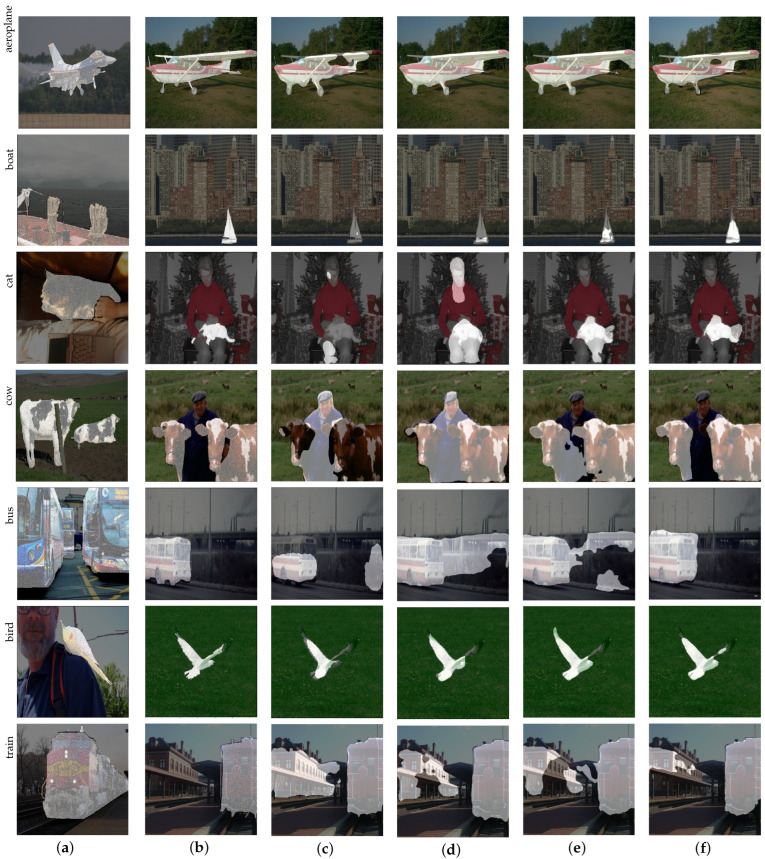
Qualitative segmentation results in 1-way 1-shot segmentation on PASCAL-$5^{i}$. Specifically, the first column is the support images with ground-truths, the second column is the query images with ground-truths, and the third, fourth, fifth, and sixth columns are the segmentation results of the query images obtained by CANet, ASGNet, PFENet, and MCEENet, respectively. (**a**) support; (**b**) ground-truth; (**c**) CANet; (**d**) ASGNet; (**e**) PFENet; (**f**) MCEENet.

Our method not only performs well on the mIoU values, but also has excellent segmentation results in actual image segmentation with only one support image. Figure 6 shows some segmentation results of MCEENet and other three state-of-the-art FSS methods: CANet, ASGNet, and PFENet. We selected these three methods because their codes are available and we can reproduce their results. As shown in Figure 6, compared with the other three methods, MCEENet can provide more accurate segmentation results, especially in some cases with complex backgrounds, which shows that the robust performance of this method is excellent. Specifically, from the comparison results of the first three rows in Figure 6, we can see that compared with the other three algorithms, the edges of the segmentation results obtained by MCEENet are more closer to the ground-truth. This proves that the proposed EAS module can guide MCEENet to segment the edge area of the objects well. From the comparison results of the latter three rows in Figure 6, we can see that MCEENet can become closer to the overall area of objects. This shows that the proposed MCE module and the parallel feature extraction network using both Vision Transformer and ResNet-50 can obtain more richer image features of the original support and query images.

### 4.5. Computational Complexity

Our experiments were conducted on an NVIDIA GTX Titan XP GPU. The training of MCEENet for 200 epochs took about 50 h on all folds of the PASCAL-$5^{i}$ dataset. Since MCEENet uses both Vision Transformer and ResNet-50 for feature extraction, the training time of MCEENet is a bit longer than methods using VGG or ResNet as the backbone. After training, MCEENet took about 0.25 s per image for inference. The fast inference speed of MCEENet shows its strong applicability to few-shot semantic segmentation scenarios.

## 5. Conclusions

In this paper, we proposed a novel FSS network named MCEENet. In MCEENet, we built two parallel weight-shared feature extraction networks to extract meaningful support and query image features, respectively. Each feature extraction network consisted of a ResNet-50 and a Vision Transformer, where ResNet-50 extracted local image features and Vision Transformer captured global dependencies of the image. After each feature extraction network, we proposed the MCE module to fuse ResNet and Vision Transformer features, which used cross-scale feature fusion and multi-scale dilated convolutions to further mine and enrich the contextual information of the image. In addition, we designed the EAS module, which combined the shallow ResNet features of the query image with the edge features computed by the Sobel operator to improve the edge parts of the segmentation results. Extensive experiments were implemented on the Pascal-$5^{i}$ and the results of the 1-shot setting and the 5-shot setting on the PASCAL-$5^{i}$ dataset are 63.5% and 64.7%, which surpasses the state-of-the-art results by 1.4% and 0.6%, respectively.

Our method also has some limitations. In the process from the input of support and query image to generating the final fuse features used to upsampling the final segmentation results, we did not use additional background information, which caused our MCEENet to lose many features that are beneficial to the final segmentation. In addition, we did not simplify the backbone of MCEENet better, which made the network more learning parameters during the training process and caused the model to be not lightweight enough when used. Our future work includes two aspects. On the one hand, we plan to extend our method to exploit the background of support and query images. On the other hand, we plan to explore neural network architecture search to obtain a lighter backbone network, which can increase the real-time performance of FSS.

## Figures and Tables

**Table 1:** PASCAL-$5^{i}$ (PASCAL VOC 2012, SBD).

Dataset	Test Classes
Fold-0	aeroplane, bicycle, bird, boat, bottle
Fold-1	bus, car, cat, chair, cow
Fold-2	dining table, dog, horse, motorbike, person
Fold-3	potted plant, sheep, sofa, train, tv/monitor

**Table 2:** Ablation study on Vision Transformer, the MCE module, and the EAS module in terms of mIoU.

Methods	1-Shot	5-Shot
MCEENet without Vision Transformer	62.6	63.2
MCEENet without the MCE modules	61.3	62.6
MCEENet without the EAS module	63.1	64.2
MCEENet	63.5	64.7

**Table 3:** Results of MCEENet and other state-of-the-art FSS methods on four folds of the PASCAL-$5^{i}$ dataset in terms of mIoU. The highest performance in each column is highlighted in boldface.

		1-Shot					5-Shot				
**Methods**	**Backbone**	**Fold-0**	**Fold-1**	**Fold-2**	**Fold-3**	**Average**	**Fold-0**	**Fold-1**	**Fold-2**	**Fold-3**	**Average**
OSLSM [13] (BMVC’18)	VGG-16	33.6	55.3	40.9	33.5	40.8	35.9	58.1	42.7	39.1	44.0
FWB [15] (ICCV’19)		47.0	59.6	52.6	48.3	51.9	50.9	62.9	56.5	50.1	55.1
PANet [14] (ICCV’19)		42.3	58.0	51.1	41.2	48.1	51.8	64.6	59.8	46.5	55.7
SG-One [30] (TCYB’20)		42.2	58.4	48.4	38.4	46.3	41.9	58.6	48.6	39.4	47.1
CRNet [41] (CVPR’20)		−	−	−	−	55.2	−	−	−	−	58.5
FSS-1000 [52] (CVPR’20)		−	−	−	−	−	37.4	60.9	46.6	42.2	56.8
HSNet [51] (ICCV’21)		59.6	65.7	59.6	54.0	59.7	64.9	69.0	64.1	58.6	64.1
CANet [38] (CVPR’19)	ResNet-50	52.5	65.9	51.3	51.9	55.4	55.5	67.8	51.9	53.2	57.1
PFENet [39] (TPAMI’20)		61.7	69.5	55.4	56.3	60.8	63.1	70.7	55.8	57.9	61.9
CWT [35] (ICCV’21)		56.3	62.0	59.9	47.2	56.4	61.3	68.5	68.5	56.6	63.7
SCL_PFENet [40] (CVPR’21)		63.0	70.0	56.5	57.7	61.8	64.5	70.9	57.3	58.7	62.9
ASGNet [31] (CVPR’21)		58.8	67.9	56.8	53.7	59.3	63.7	70.6	64.1	57.4	63.9
SAGNN [34] (CVPR’21)		64.7	69.6	57.0	57.3	62.1	64.9	70.0	57.0	59.3	62.8
MCEENet	ResNet-50 and Vision Transformer	64.8	73.0	59.4	57.0	63.5	66.4	73.8	60.0	58.8	64.7

**Table 4:** Results of MCEENet and other state-of-the-art FSS methods on four folds of the PASCAL-$5^{i}$ dataset in terms of FB-IoU. The highest performance in each column is highlighted in boldface.

		FB-IoU (%)	
**Methods**	**Backbone**	**1-Shot**	**5-Shot**
Co-FCN [30]	VGG-16	60.1	60.2
SG-One [30]		63.1	65.9
PANet [14]		68.5	70.7
CANet [38]	ResNet-50	66.2	69.6
ASGNet [31]		69.2	74.2
PGNet [32]		69.9	70.5
SAGNN [34]		73.2	73.3
PFENet [39]		73.3	73.9
MCEENet	ResNet-50 and Vision Transformer	77.0	77.8

## Data Availability

Not applicable.
